# Inter- and Intraobserver reproducibility of T2 relaxation times of the discus interpubicus: A feasibility study at 3 Tesla

**DOI:** 10.1371/journal.pone.0202698

**Published:** 2018-08-22

**Authors:** Kai-Jonathan Maas, Maxim Avanesov, Azien Laqmani, Julius Weinrich, Markus Sauer, Michael G. Kaul, Gerhard Adam, Marc Regier, Cyrus Behzadi

**Affiliations:** Department of Diagnostic and Interventional Radiology and Nuclear Medicine, University Medical Center Hamburg-Eppendorf, Hamburg, Germany; Johns Hopkins School of Medicine, UNITED STATES

## Abstract

**Objective:**

To quantify standard values of the discus interpubicus in healthy subjects and to determine reliability and repeatability using T2 relaxation time measurements at 3T.

**Methods:**

20 asymptomatic participants (10 male, 10 female; mean age: 27.3 years ±4.1, BMI: 22.2 ±1.8) underwent a 3T Magnetic Resonance Imaging (MRI) of the pelvic region in a supine position. We included sagittal and para-axial T2w sequences centred over the pubic symphysis in order to identify the complete discus interpubicus. For quantitative analysis, a multi-echo Turbo Spin Echo (TSE) sequence (including 12 echo times between 6.4 and 76.8 ms) was acquired and analysed by using an in-house developed quantification plugin tool (qMapIt) extending ImageJ. Two readers in consensus defined three central slices of the pubic symphysis with the greatest length. For each slice, both readers separately placed three regions-of-interest (ROI) covering the whole discus interpubicus. Both readers repeated the ROI placements in identical fashion after a four-week interval on the original MRI images. Statistical analysis included intraclass correlation coefficient (ICC), nonparametric Wilcoxon test, Fisher exact test and mean relaxation time in ms and 95% confidence intervals.

**Results:**

T2 relaxation time analysis was performed for all 20 participants. In total, a mean relaxation time of all analysed segments for both observers was 48.6 (±6.3 ms), with a mean relaxation time for observer 1 of 48.7 (±6.0 ms) and for observer 2 of 48.5 ms (±6.6ms). The calculated ICC comparing inter- and intrarater reproducibility was excellent in all segments (≥0.75).

**Conclusion:**

T2 mapping of the discus interpubicus demonstrates good inter- and intrarater repeatability as well as reliability. Mean relaxation times were calculated with 48.6ms in healthy volunteers.

## Introduction

Osteitis pubis (OP) is considered as one of the major reasons for groin pain in athletes overall [1]. It is reported that a time span of more than 12 months is needed until clinical symptoms disappear and the athlete can return to play [2]. Athletes performing rapid changes in direction and side to side moves in high level pivoting sports are especially prone to injuries of the pubic symphysis [3]. In soccer players for example, up to 13% of all injuries are linked to the groin area [4].

The pubic symphysis is an amphiarthrodial joint situated between the pubic bones. The discus interpubicus is centrally located in the pubic symphysis between the joint surfaces of the pubic bones (Facies symphysialis) while representing an important stabilizer for biomechanical shear forces. It consists of a thick, fibrocartilaginous disc which blends into the surrounding ligamentous structures and a thin cartilage layer of the articular surfaces of the pubic bones [5]. Four ligamentous structures surround the pubic symphysis compensating for a lack of joint capsule. At the cranial aspect of the discus interpubicus attaches the superior pubic ligament, which connects the pubic tubercles. At the caudal aspect of the discus interpubicus is the inferior (= arcuate) pubic ligament situated in the angulus subpubicus. The former mentioned thick arcuate ligament is considered as the most important mechanical stabilizer of the pubic symphysis[6]. It also blends into the aponeurosis of the gracilis and adductor longus muscles. In addition, the pubic symphysis is also surrounded by an anterior and posterior ligament attached to the discus interpubicus [6]. The anterior ligament blends into the fascia of the musculus rectus abdominis and the posterior ligament into the fascia of the abdominal wall. The discus is also surrounded by various muscle insertions originating from the upper and lower body as e.g. the musculus rectus abdominis and the adductor muscles. This anatomic constellation demonstrates the enormous mechanical forces centred on the pubic symphysis. MRI is doubtlessly considered as a reference standard for investigating joint structures. However, although image resolution has constantly improved due to recent developments in scanner hardware and coils used as receivers, the detection of initial cartilage damage remains challenging e.g. in cases of thin cartilage layers and narrow joint spaces [7]. In the last decade, several quantitative techniques like T2, T2* or T1rho have proven their ability at detecting compositional changes in articular cartilage[8–10]. It could be demonstrated that proteoglycan (PG) and glycosaminoglycan (GAG) levels in articular cartilage are reduced in initial stages of osteoarthritis (OA) prior to morphological manifestations. Therefore, these techniques are considered as promising non-invasive tools for detecting initial stages of OA. Especially knee joint cartilage has been investigated in several publications [11,12]. Recent studies further elaborated on these validated techniques by transferring them to different anatomical regions. For example, joints with rather thin cartilage layers such as the hip [13,14] or the wrist [15,16] have been successfully investigated. In one publication, the authors even successfully demonstrated the feasibility of T2 relaxation time measurements of the sacroiliac joint in a preliminary study design demonstrating moderate inter- and intraobserver reliability[17].

Additional knowledge of the pubic symphysis is of high clinical interest. Especially in athletes with unclear groin pain and e.g. inconclusive morphologic MRI results, quantitative measurements might help identifying the accurate diagnosis at an early time point.

Therefore the aim of our study was to investigate the pubic symphysis with T2 relaxation time measurements at 3T in order to quantify standard values of the discus interpubicus in healthy subjects and to determine reliability and repeatability.

## Methods

The study was approved by the Clinical Institutional Review Board (Ethic committee of the medical chamber of Hamburg) and the background of the study was explained to all participants. Prior to MRI, written informed consent from all participants was given.

We investigated 22 young adults (12 male, 10 female participants; mean age 27 ±3.9 years) without any known prior history of pelvic pain or clinical signs suggestive of pubic osteitis. Two male participants had to be excluded from the final study group due to motion artifacts in the multi-echo sequence. Therefore, finally 20 healthy volunteers were included in this prospective study consisting of 10 male (mean age 29.3 ±3.3 years, mean BMI 22.1 ±1.8) as well as 10 female participants (mean age 25.2 ±3.8 years, mean BMI 21.4 ±1.8). Previous pregnancy was ruled out in all female participants for it might have confounded quantitative analysis of the discus interpubicus. All participants were asked not to perform in any physical activity for up to 24 hours prior to the MRI scan and only participants not exceeding a regular physical activity of more than three hours per week were included. At the time of MRI examination, no participant reported pain in the pelvic region or history of major trauma or surgery of the pubic symphysis. The morphological sequences were read by one senior radiologist in order to identify motion artifacts or imaging abnormalities suspicious of e.g. signs of damage to the pubis symphysis and systemic diseases such as irregular widening, posttraumatic tears of the discus or congenital anomalies. A commercially available post-processing workstation (Intelligence workspace, Philips) was used for image analysis.

### Image acquisition

The same 3T MRI scanner (Ingenia, Philips, Best, the Netherlands) was used for all participants. Individuals were positioned in a supine position and a knee wedge was placed under the patients popliteal fossa with a maximum flexion of 10°. We used a 32-channel anterior body coil with 60 cm coverage centred over the participants pelvic region. After the survey scan was acquired a 15 seconds standard B1 inhomogeneity correction scan was performed. Our imaging protocol is outlined in detail in Table 1.

**Table 1:** Imaging parameters.

Sequence parameters	Survey	T2w TSE	T2w TSE	Multi-echo TSE
Excitation pulse	2D	2D	2D	2D
Orientation	Axial, sagittal, coronal	Sagittal	Para-axial	Para-axial
TR (milliseconds)	4.5	3239	2148	1145
TE (milliseconds)	2.3	105	105	12 (6.4, 12.8, 19.2, 25.6, 32, 38.4, 44.8, 51.2, 57.6, 64, 70.4, 76.8)
Flip angle (°)	25	90	90	90
Turbo factor		26	31	12
SENSE factor	-	-	1	2
Averages	1	1.5	2	2
Field of View (mm)	450	121	150	160
Matrix	256 x 128	136 x 136	188 x 184	212 x 213
Slices	3 x 5	23	13	9
Slice thickness (mm)	15	3	3	3
Gap (mm)	10	0	0	0
Voxel size (mm^3^)	1.76 x 3.52 x 15	0.9 x 0.85 x 3	0.8 x 0.8 x 3	0.75 x 0.75 x 3
Scan time (min)	0:07	2:48	1:52	11:36

Imaging parameters for all sequences performed including multi-echo TSE sequence resulting in a total scan time of approximately 16 minutes.

**Fig 1:**
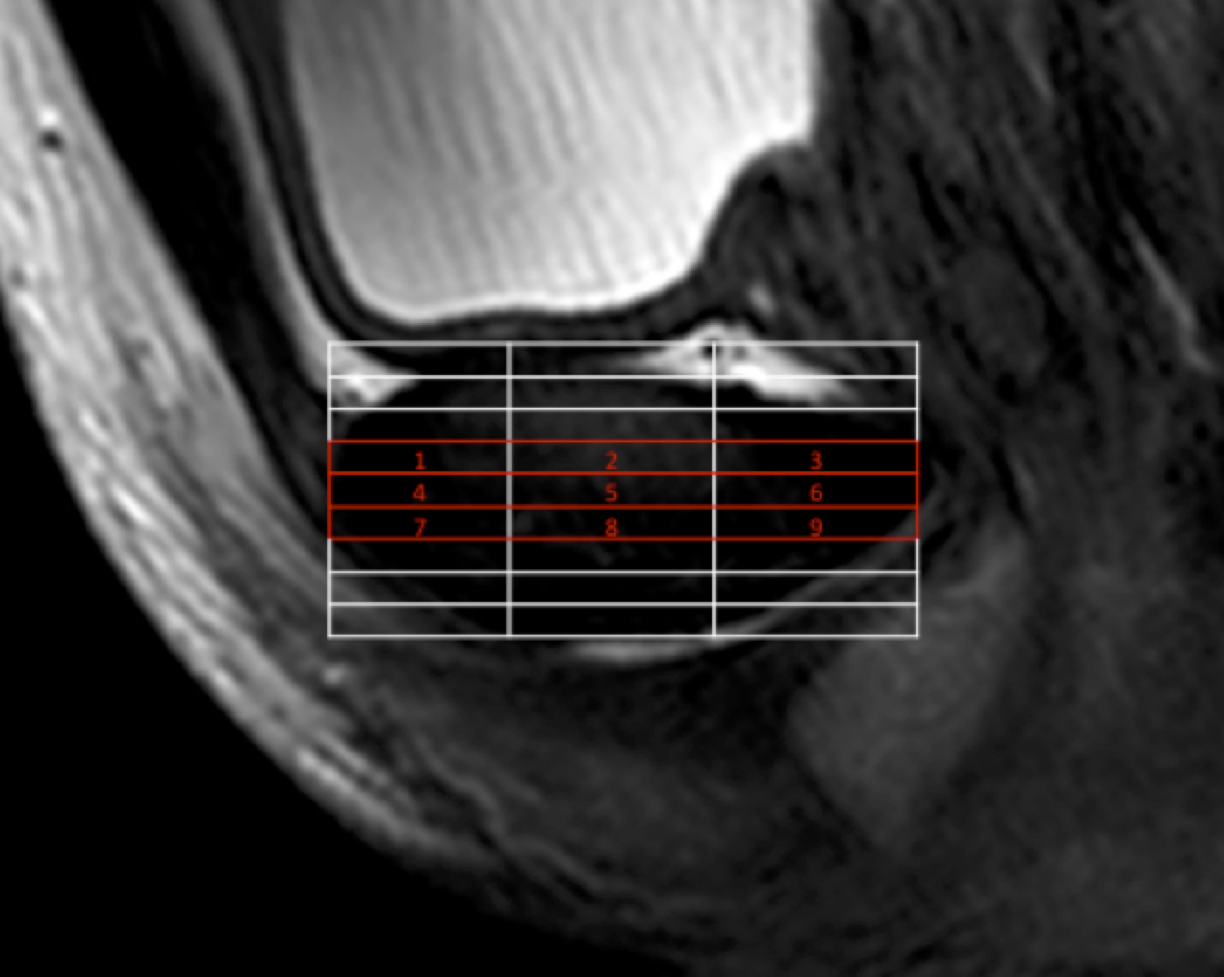
Sagittally acquired T2w TSE sequence was used for anatomic identification of the pubic symphysis. Afterwards a multi-echo sequence was planned parallel to the long-axis of the symphysis (white and red box). For quantitative analysis, the three central slices of the symphysis (red) were determined and in each slice three segments analysed (Segment 1 to 9).

It consisted of a sagitally acquired T2w sequence centred over the pubic symphysis and was aligned parallel to the arcuate line. This T2w sequence was used for identification of the discus interpubicus (Fig 1).

**Fig 2:**
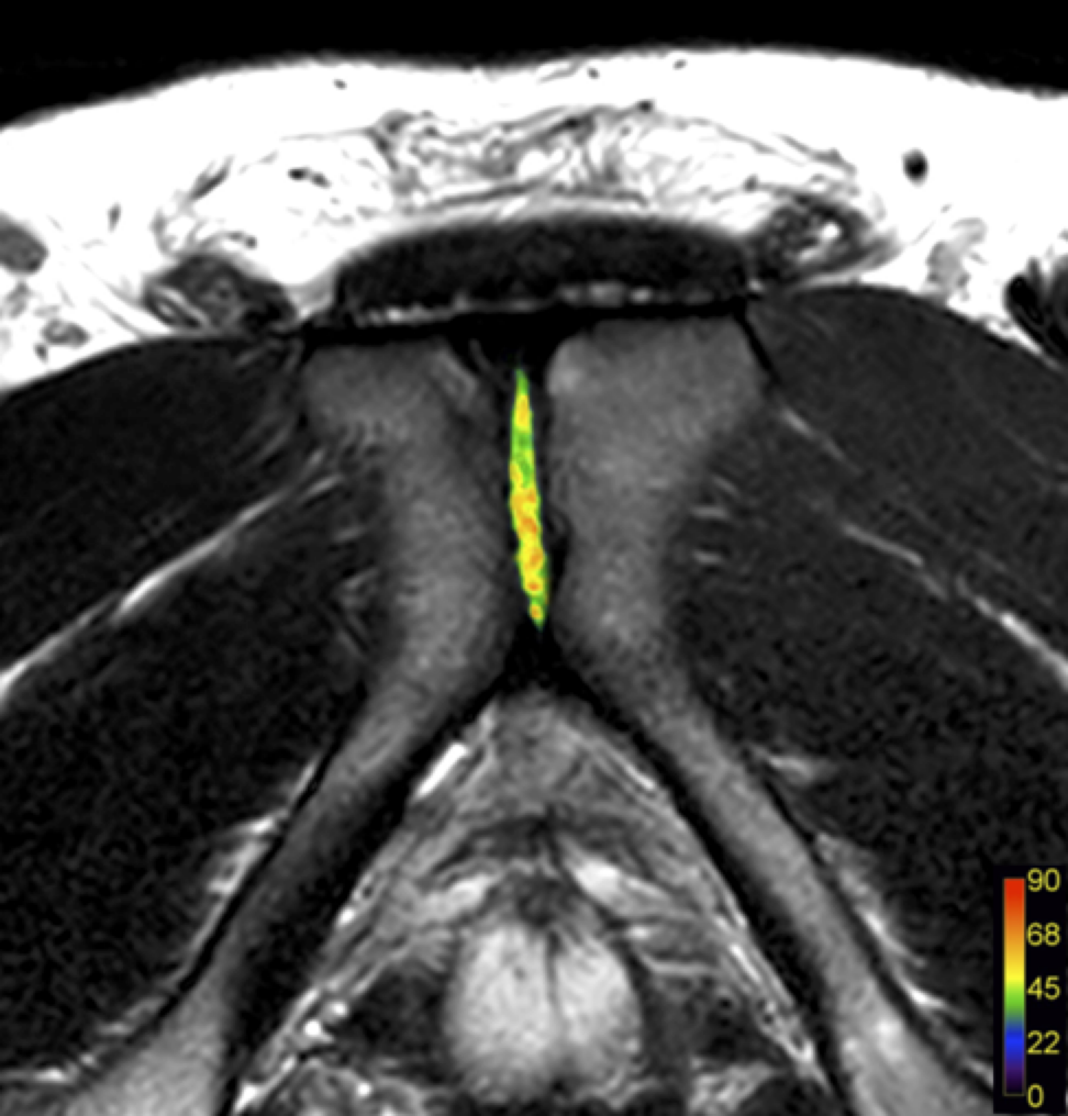
Multi-echo overlay of the discus interpubicus and colour bar (in milliseconds) in one participant demonstrating homogenic relaxation times.

Aligned to the central axis of the long axis of the pubic symphysis, we afterwards acquired a T2w para-axial sequence, which was angulated horizontally to the pubic symphysis including its full extent. After visual confirmation of including the full pubic symphysis, a multi-echo Turbo Spin Echo (TSE) sequence (including 12 echo times between 6.4 and 76.8 ms) was acquired in identical orientation to the afore mentioned para-axial T2w sequence (Fig 2).

By using an in-house quantification plugin tool (qMapIt) extending ImageJ (National Institutes of Health, Bethesda, MD), we performed T2 mapping by fitting a monoexponential function A*exp(–TE/T2) to the multiecho data with A being an amplitude. The first echo was neglected because it does not contain any signal of a stimulated echo in contrast to the later ones.

### Quantitative analysis

Two radiologists (6 and 12 six years of experience in quantitative measurements) with special interest in musculoskeletal imaging performed the quantitative image analysis. They consensually defined the three central slices of the pubic symphysis with the greatest length in each participant. Any further slices were excluded from our analysis in order to prevent inconsistent measurements due to partial volume effects. Each observer individually divided the discus interpubicus on the three predefined slices into three segments of identical size. Afterwards, the observers separately placed in each segment one Region-of-interest (ROI) on the discus interpubicus resulting in 3 ROIs of identical size in each slice (9 in every participant). Special attention was paid to exclude any surrounding tissue such as the pubic bone or attaching ligamentous structures. After a four-week interval, both radiologists repeated ROI placements in an identical fashion in all participants on the original data sets. In the end both observers had placed 720 ROIs. For each ROI, mean relaxation time in ms and confidence intervals were noted.

### Statistical analysis

Data collection and statistical calculations were performed by using SPSS Statistics 22 software (IBM Inc. SPSS Statistics, Chicago, IL) and MedCalc 15.8 (MedCalc Software, Ostend, Belgium).

The intraclass correlation coefficient (ICC) was calculated in order to compare inter- and intrarater reproducibility of ROI placements. An ICC of less than 0.4 was considered as poor, 0.4–0.75 as fair to good and an ICC of 0.75 or higher as excellent in accordance to previously published data[18]. Normal distribution of data samples was evaluated and proven by Shapiro-Wilk test. Mean differences between the two observers and between two measurements of the same observer were analysed by using a nonparametric Wilcoxon test. Variances between two data samples were compared by the F test. Bland-Altman analyses was used to assess agreement between inter- and intraobserver measurements. Group comparisons of continuous variables between three groups were performed using Kruskal-Wallis test. Subsequent pairwise post-hoc analysis was performed by the Wilcoxon signed-rank test. Statistical significance was defined as p<0.05 and was adjusted to p<0.017 using the Bonferroni correction to adapt for multiple comparisons as appropriate.

## Results

In the 20 included data sets acquired between January and May 2018, margins of the pubic symphysis could be easily detected and the central slices aspect of the discus interpubicus defined.

Our statistical evaluation including ICC is displayed in detail in Table 2. In total, mean relaxation time of all analysed segments for both observers was 48.6 (±6.3 ms). The first time the data was analysed, a mean T2 relaxation time of 48.7 ms (±6.1 ms) was noted for observer 1. For observer 2 a mean relaxation time of 48.6 ms (±6.6ms) was found. After a four-week time interval, mean relaxation time for observer 1 was calculated to be 48.8 ms (±5.9 ms) and 48.4 ms (±6.7 ms) for observer 2. In summary, mean relaxation time was calculated to be 48.7 (±6.0 ms) for observer 1 and 48.5 ms (±6.6 ms) for observer 2. The indexes of interobserver reliability and intraobserver reproducibility for relaxation time measurements in each defined cartilage segment were all distinctly greater than 0.75. These findings indicate a very good interobserver reliability and intraobserver reproducibility (Table 2). The mean differences for observer 1 comparing measurements between the two repeated analyses ranged from -0.27 to 0.91 ms (SD: 0.98–3.1). For observer 2, mean differences were noted from -0.28 to 0.5 ms (SD: 1.3–2.0). Statistical analysis (nonparametric Wilcoxon and Fisher exact test) revealed that mean differences for inter- and intrarater comparison were all insignificant (p>0.05). In an additional analysis comparing mean relaxation times of the predefined segments (1/4/7 vs. 2/5/8 vs. 3/6/9) insignificant differences were noted (p>0.05). Comparing mean relaxation times of each defined slice (upper, central, lower), a significant difference was detected after performing Bonferroni correction (p<0.017) between the upper and central slice. Insignificant differences were noted between the upper and lower as well as the central and lower slice.

**Table 2:** Inter- and intraobserver agreement.

**Interobserver agreement**									
	Slice 1			Slice 2			Slice 3		
	1	2	3	4	5	6	7	8	9
**Observer 1**									
Mean difference	-0.27	0.26	0.40	0.91	0.17	0.21	-0.12	0.33	0.38
SD	0.98	1.11	1.21	2.47	1.95	1.14	3.11	1.00	1.95
Variance	0.97	1.23	1.47	6.12	3.82	1.31	0.67	1.00	3.82
Limits of agreement	-1.66 to 2.19	-1.91 to 2.43	-1.98 to 2.77	-3.94 to 5.76	-3.66 to 3.99	-2.04 to 2.45	-6.22 to 5.97	-1.63 to 2.30	-3.45 to 4.21
ICC (95%CI)	0.99 (0.98–1.00)	0.99 (0.99–1.00)	0.99 (0.98–1.00)	0.98 (0.95–0.99)	0.98 (0.96–0.99)	0.99 (0.98–1.00)	0.97 (0.92–0.99)	0.99 (0.99–1.00)	0.98 (0.96–0.99)
P-value (Wilcoxon test)	0.33	0.26	0.18	0.11	0.57	0.45	0.78	0.13	0.43
P-value (F-test)	0.99	0.91	0.89	0.61	0.78	0.98	0.94	1.00	0.73
**Observer 2**									
Mean difference	-0.02	-0.03	-0.28	0.01	-0.04	-0.24	-0.28	0.39	0.47
SD	1.35	1.86	1.47	1.99	1.56	1.39	1.32	2.02	1.89
Variance	1.83	3.47	2.17	3.98	2.44	1.94	1.74	4.08	3.56
Limits of agreement	-2.66 to 2.63	-3.69 to 3.62	-3.17 to 2.60	-3.90 to 3.91	-3.20 to 3.02	-2.97 to 2.49	-2.87 to 2.30	-3.56 to 4.35	-3.23 to 4.17
ICC (95%CI)	0.99 (0.97–1.00)	0.99 (0.96–0.99)	0.99 (0.97–1.00	0.99 (0.97–0.99)	0.99 (0.97–1.00)	0.99 (0.97–0.99)	0.99 (0.98–1.00)	0.98 (0.95–0.99)	0.98 (0.95–0.99)
P-value (Wilcoxon test)	0.47	0.84	0.33	0.65	0.62	0.41	0.31	0.68	0.50
P-value (F-test)	0.93	0.91	0.98	0.95	0.89	0.89	0.85	0.87	0.88
**Intraobserver agreement**									
	Slice 1			Slice 2			Slice 3		
	1	2	3	4	5	6	7	8	9
Mean difference	0.18	-0.35	-0.58	-0.28	-0.29	0.38	-0.13	-0.18	0.27
SD	1.06	1.75	2.25	1.71	1.76	1.22	2.37	2.57	1.27
Variance	1.13	3.06	5.05	2.91	3.10	1.49	5.61	6.60	1.62
Limits of agreement	-1.90 to 2.27	-3.78 to 3.07	-4.99 to 3.83	-3.62 to 3.07	-3.74 to 3.16	-2.01 to 2.77	-4.77 to 4.52	-5.21 to 4.86	-2.23 to 2.76
ICC (95%CI)	0.99 (0.98–1.00)	0.99 (0.99–1.00)	0.97 (0.93–0.99)	0.99 (0.98–1.00)	0.99 (0.97–0.99)	0.99 (0.97–1.00)	0.98 (0.95–0.99)	0.97 (0.93–0.99)	0.99 (0.98–1.00)
P-value (Wilcoxon test)	0.31	0.55	0.67	0.35	0.67	0.23	0.45	0.50	0.50
P-value (F-test)	0.91	0.98	0.82	0.96	0.97	0.87	0.97	0.80	0.99

Detailed overview of the performed statistical analysis in nine segments in three consecutive slices for the two observers including inter- and intrarater agreement.

## Discussion

In this study we could successfully demonstrate the feasibility of quantitative imaging of the pubic symphysis in 20 healthy volunteers at 3T MRI. Quantitative analysis was based on a sagittally acquired T2w TSE sequence orientated parallel to the long axis of the symphysis and perpendicular to the arcuate line as previously reported for morphological analysis[5]. After recognition of the central aspect of the symphysis, a para-axial multi-echo TSE sequence parallel to the long axis of the pubic symphysis was acquired for quantitative analysis. Our results demonstrate a constantly high inter- and intrarater reproducibility. In all predefined segments, mean differences between the two observers as well as for each observer comparing two measurements with a four-week interval were insignificant (p>0.05).

T2 relaxation time analysis has proven its capability of analysing articular cartilage in many publications. Particularly knee joint cartilage has been investigated in several studies[12,19,20]. By using this technique, initial stages of cartilage degeneration could be detected prior to morphological manifestations e.g. in patients after anterior cruciate ligament (ACL) reconstruction[21,22]. Due to its benefits, quantitative analysis has been used recently in different joints such as the shoulder[23] or the ankle[24,25]. For example the authors calculated a mean T2 relaxation time for the tibiotalar joint in healthy female volunteers between 27.7 and 40.6 ms[25], 38 ms for glenoid cartilage[23] and up to 116 ms for intervertebral discs in male participants[26]. In our analysis of the pubic symphysis, an overall mean relaxation time of 46.77 ms (±6.8ms) for the two observers was detected at 3T. Validity and reliability of our measurements are proven by insignificant intra- and interrater differences. In all segments a high ICC conforming our ROI placements between the two time points as well as between the two observers was monitored. Therefore we think that the acquisition of T2 mapping can be an additional tool in the detection and exclusion of pathologies of the discus interpubicus, because the correct identification of especially osteitis pubis based on morphological imaging is often challenging [27]. Additional information due to technical improvements such as quantitative imaging is desirable. Therefore we consider athletes suffering from long-term indistinct groin pain a potential clinical indication for performing additional T2 mapping. Quantitative analysis might be performed prior to e.g. invasive diagnostic procedures such as fluoroscopy-guided symphyseal injection of a contrast media with potential risk factors such as pain or infection. We particularly ascribe a high clinical impact on a consecutive study design analysing T2 relaxation times in athletes with indistinct groin pain. These patients might benefit from correct identification of the source of pelvic pain based on quantitative imaging at an earlier time-point accelerating return-to-play.

We would like to address several limitations of our preliminary investigation. As a major limitation, it has to be mentioned that no standard of reference such as a histological correlation was performed in order to verify our findings. The primary focus of our investigation represents the quantitative analysis of the pubic symphysis based on a high-resolution multi-echo sequence with a small pixel size. With an acquisition time of 11:36 minutes, the presence of motion artifacts has to be concerned. For clinical application, a shorter acquisition time as well as a potential use of T2 mapping at 1.5T are desirable while maintaining the same reliability. This problem might be solved by focusing the quantitative analysis on the central aspect of the pubic symphysis or by reducing pixel resolution. A reduced pixel resolution would increase the signal-to-noise ratio so that a higher SENSE factor could be applied to further shorten the scan time. Nonetheless, at a lower field strength of 1.5 T accompanied with an adjusted pixel resolution, subtle pathologies in thin anatomic structures might be missed due to inaccurate ROI placements. Especially in the analysis of the discus interpubicus reduced pixel resolution might confound image analysis if the boundaries to the adjacent hyaline cartilage are not as exact due to degenerative changes. However, the general identification of pathologies of the discus interpubicus seems feasible at 1.5T. It would be interesting to identify if there might be an analogous trend in patients with high physical activity of the pelvis e.g. soccer players, leading to altered relaxation times as shown for articular cartilage of the tibiotalar joint[28]. In the investigated cohort of asymptomatic patients, no existing injuries were detected e.g. longitudinal clefts of the discus interpubicus or edema of the pubic symphysis, which might theoretically bias correct quantitative analysis. However, we believe that these conflicts are less serious when compared to different joints being investigated with relaxation time measurements. For instance, the recently evaluated sacroiliac joint is prone to inconsistent quantitative measurements due to the common presence of the vacuum phenomena in 34% of all investigated patients [29]. Apart from the above mentioned limitations, we would like to address our rather small study cohort of 20 participants. Nonetheless, T2 relaxation times were measured twice in each participant. As a final limitation we like to point out that our study demonstrates a high intra- and interrater reproducibility of ROI based T2 mapping. However, for MRI scans were not repeated in the same individuals or phantoms at various time points, the reproducibility of the performed multi-echo sequence cannot be postulated based on our study design. This question could be answered e.g. in a longitudinal setting including repeating T2 mapping of the discus interpubicus.

## Conclusion

In our feasibility study, T2 mapping of the discus interpubicus demonstrates good inter- and intrarater repeatability as well as reliability in healthy volunteers.

## Supporting information

S1 Dataset (XLSX)
